# ZBTB7A, a potential biomarker for prognosis and immune infiltrates, inhibits progression of endometrial cancer based on bioinformatics analysis and experiments

**DOI:** 10.1186/s12935-020-01600-5

**Published:** 2020-11-09

**Authors:** Rong Geng, Yuhua Zheng, Donghua zhou, Qingdong Li, Ruiman Li, Xiaoling Guo

**Affiliations:** 1 Department of Gynecology, Affiliated Foshan Maternity & Child Healthcare Hospital, Southern Medical University, Foshan, 52800 China; 2 Department of Gynecology and Obstetrics, The First Affiliated Hospital, Jinan University, Guangzhou, 510632 China; 3 Foshan Maternal and Children Healthy Research Institute, Affiliated Foshan Maternity & Child Healthcare Hospital, Southern Medical University, Foshan, China; 4 Department of Pathology, Affiliated Foshan Maternity & Child Healthcare Hospital, Southern Medical University, Foshan, China

**Keywords:** ZBTB7A, Uterine corpus endometrial carcinoma, Prognostic biomarker, Immune cell infiltration, E2F4

## Abstract

**Backgroud:**

ZBTB protein is an important member of the C2H2 zinc finger protein family. As a transcription factor, it is widely involved in the transcriptional regulation of genes, cell proliferation, differentiation, and apoptosis. The ZBTB7A has been largely linked to different kinds of tumors due to its diverse function. However, the value for ZBTB7A in uterine corpus endometrial carcinoma (UCEC) is unclear.

**Methods:**

In our work, we assessed the importance of ZBTB7A in UCEC. Firstly, Using Oncomine and Tumor Immunoassay Resource (TIMER) databases to evaluate the expression of ZBTB7A. Secondly, we explored the co-expression network of ZBTB7A through the cBioPortal online tool, Metascape, and LinkedOmics. TIMER was also used to explore the relationship between ZBTB7A and tumor immune invasion, and to detect the correlation between the ZBTB7A and the marker genes related to immune infiltration. Finally, CCK8, migration, ChIP assays were introduced to partly validate ZBTB7A function in endometrial cancer cells.

**Results:**

We found the ZBTB7A expression in TIMER was associated with various cancers, especially UCEC. The decreased expression of ZBTB7A was markedly related to the stage and prognosis of UCEC. Furthermore, ZBTB7A was also related to the expression of various immune markers such as Neutrophils, Dendritic cell, T cell (general), Th1, Th2, and Treg. Finally, we verified that ZBTB7A repressed E2F4 transcription and inhibited cells proliferation and migration. These results indicate that ZBTB7A may play a vital role in regulating immune cell infiltration in UCEC, and is a valuable prognostic marker.

**Conclusions:**

In summary, we demonstrate that ZBTB7A is notably downregulated in UCEC, plays a vital role in regulating immune cell infiltration, possesses diagnostic and prognostic values and attenuates E2F4 transcription and cell proliferation, migration in vitro.

## Background

Uterine corpus endometrial carcinoma (UCEC) is the sixth-most-common type of cancer in females, and the incidence is rapidly increasing in recent years [1]. The 2018 data shows that 382,000 women are diagnosed and 90,000 deaths due to UCEC worldwide [2]. The factors that affect endometrial cancer are diverse. First, the instability of estrogen levels is one of the most common causes, so postmenopausal women are more susceptible to endometrial cancer [3]. Besides, many factors, including smoking, hypertension, overweight, and genetics, play a vital role in the development of endometrial cancer [4]. Despite progress in the treatment of endometrial cancer, endometrial cancer is one of the human malignant diseases for which mortality is increasing. Unexpected recurrences and poor prognosis still confuse clinicians. Therefore, it is an urgent need to detect more therapeutic targets and prognostic markers.

ZBTB7A is also called Pokemon, factor binding IST protein-1(FBI-1), and the lymphoma/leukemia-related factor (LRF). ZBTB7A is overexpressed in many human cancers, including ovarian [5], breast [6], as an oncogene. Further research on the carcinogenic function of ZBTB7A was found that ZBTB7A could affect the survival, proliferation, apoptosis, and migration of cancer cells. Besides, the expression of ZBTB7A is also related to many clinicopathological parameters, including tumor size, histological grade, and overall patient survival. ZBTB7A regulates gene expression by transducing other transcription factor signals and is a widely used as promoter factor [7]. In breast cancer, ZBTB7A controls the expression of ERα by directly binding the GC-rich region in the ERα promoter [8]. In hepatocellular carcinoma, ZBTB7A increases the expression of P53 and initiates caspase-dependent apoptosis through death receptors and mitochondrial pathways [9]. Recent studies have shown that ZBTB7A is closely related to the tumor microenvironment and immune cell infiltration in prostate cancer [10]. It is worth notable that the chromosomal region 19p13.3, including ZBTB7A, is often deleted in human cancer. The loss of ZBTB7A may be one of the significant potential mechanisms that causes cancer [11]. However, the expression pattern and its significance in UCEC have not been well identified.

Our results indicate that ZBTB7A may be a new potential prognostic marker in endometrial cancer. We analysed the expression, mutation and prognosis of ZBTB7A in UCEC patient via Oncomine, GEPIA, TIMER, and cBioPorta database. The relation of ZBTB7A and immune cell infiltration was further investigated through TIMER. Moreover, we expanded discussion to see if the association holds up in pan-carcinomas. Finally, biomedical experiments, such as CCK8, migration and ChIP assays were carried out to validate conclusion from bioinformatic analysis partly. Therefore, our results reveal ZBTB7A as a new target and direction for the diagnosis and treatment of endometrial cancer.

## Materials and methods

### Oncomine database analysis

The expression level of the ZBTB7A gene in UCEC was estimated in the Oncomine 4.5 database (https://www.oncomine.org/). Oncomine is a comprehensive database related to cancer assessment. The threshold was determined according to the following values: P-value of 0.01, fold change of 2, and gene ranking of all.

### GEPIA database analysis

The Gene Expression Profiling Interactive Analysis (GEPIA) database (https://gepia.cancer-pku.cn/) is an online database that includes 9,736 tumors and 8,587 normal samples from TCGA and the GTEx projects [12]. We applied GEPIA to evaluate the expression and survival of ZBTB7A, including overall survival (OS) and disease-free survival (DFS) based on gene expression with the log-rank test and the Mantel-Cox test in UCEC.

### UALCAN database analysis

UALCAN (https://ualcan.path.uab.edu) uses TCGA level 3 RNA-seq and clinical data from 31 cancer types [13]. UALCAN was used to analyze the relative gene expression of tumor and normal samples, as well as the gene expression of different tumor subgroups. In addition, based on individual cancer stage, tumor grade, or other clinicopathological features, UALCAN can also be used for analysis.

### cBioPortal database analysis

The cBio Cancer Genomics Portal (https://cbioportal.org) has multidimensional cancer genomics datasets [14]. The mutation of ZBTB7A in UCEC was analyzed using the cBioPortal tool in this study.

### LinkedOmics database analysis

The LinkedOmics database (https://www.linkedomics) is mainly used to analyze 32 TCGA cancer-related data sets and is a comprehensive online platform [15]. We analyzed the co-expression of ZBTB7A on the volcano map, heat map, or scatter map through the LinkedOmics function module by using Pearson's correlation coefficient. Besides, we analyzed kinase-target enrichment, miRNA-target enrichment, and transcription factor-target enrichment through gene set enrichment analysis (GSEA). The latter two network analyses were built on the Molecular Signatures Database (MSigDB). The rank norm was FDR < 0.05, and 500 simulations were performed.

### TIMER database analysis

TIMER is a comprehensive resource for systematic analysis of immune infiltrates across diverse cancer types from TCGA (https://cistrome.shinyapps.io/timer/), which includes 10,897 samples across 32 cancer types [16]. TIMER speculates the abundance value of tumor infiltrating immune cells (TIICs) from the gene expression profile by applying the method of deconvolution [17]. We analyzed ZBTB7A and the correlation of ZBTB7A expression with the abundance of immune infiltrates, including B cells, CD4 + T cells, CD8 + T cells, neutrophils, macrophages, and dendritic cells in UCEC and other cancers.

### STRING database analysis

STRING database (version 10.5) was acquired protein–protein interaction information of DEmRNAs (https://string-db.org/). Edges with weight beyond a threshold of 0.4 were displayed. Hub genes were found by using "barplot" in the R language [18].

### Metascape database analysis

Metascape [19] (https://metascape.org), which was updated in 2018, is a web‑based tool that provides gene functional annotation and enrichment analysis. Gene enrichment was analysed from Gene Ontology (GO) and Kyoto Encyclopedia of Genes and Genomes (KEGG) through the database to predict the potential biological value of overlapping genes between target genes and DEGs.

### Cell culture

endometrial cancer cell line Ishikawa cells were cultured with RPMI-1640 Medium (Gibco, Carlsbad, USA) with 10% fetal bovine serum (Gibco) at a humidified atmosphere of 5% CO_2_ at 37 °C.

### Cell viability assay

For CCK8 assay, 1000 ZBTB7A overexpressed and control cells were planted into 96-well plates, Which were cultured for varied days (1, 2, 3 and 4 day). At the point time, CCK-8 solution (G-Clone, China) 20 μl was added to each well and nurtured for another 4 h at 37 °C. Finally, the optical density (OD) value at 450 nm was set by micro-plate reader.

### Migration assay

2 × 10^5^ Ishikawa cells per/well were seeded into the upper chamber of 8 μm pore (BD Falcon, USA). Chambers were embeded into correlating 24-well plate containing RPMI-1640 medium with 20% FBS. Cells in the upper chambers were cultured without FBS. After 16–20 h, migrated cells sticking to the lower surface of chamber were fixed and stained with 5% crystal violet and were calculated in five fields of microscope.

### Quantitative PCR

The experimental procedures were described previously [20]. The primers used were listed as follows: F: 5′-CATCCAGTGGAAGGGTGTG-3′, R: 5′-TTGGACGTGAGGCTTCCTG-3′ (E2F4); F: 5′-TGCAAGGTCCGCTTCACCAG-3′, R: 5′-TGCAAGGTCCGCTTCACCAG-3′ (ZBTB7A). F: 5′-CTCCTCCTGTTCGACAGTCAGC-3′, R: 5′-CCCAATACGACCAAATCCGTT-3′(GAPDH). qPCR Parameters were confirmed as below: 95 °C for 15 s; 95 °C for 5 s, 60 °C for 40 s for 40 cycles; 95 °C for 15 s, 60 °C for 1 min and 95 °C for 15 s.

### Luciferase assays

The three deleted potential ZBTB7A binding motif of E2F4 (− 483 ~ − 471, − 45 ~ − 43, + 9 ~ + 21) and full length of E2F4 promoter (− 2000 ~ + 100) were constructed into pGL3-based vector. Cells were co-transfected with corresponding constructed pGL3 reporter plasmid and pcDNA4-ZBTB7A using Lipofectamine 2000 (Invitrogen, Carlsbad, CA, USA). After transfection 36 h, the cells were collected and measured by Dual-Luciferase Reporter assay (Promega, Madison, WI, USA). All plasmids were synthesized by the Genewiz Company (Suzhou, China).

### Chromatinimmunoprecipitation (ChIP) assay

ChIP assays were performed via ChIP Kit (Beyotime Shanghai, China). Briefly, the protein/DNA complexes were prepared 48 h from Ishikawa cells. Crosslinking of proteins to DNA was fixed using 1% formaldehyde for 10 min. The DNA were sheared to 200–1000 bp lengths sonically on ice. The lysates were slow whirling overnight at 4 °C with anti-ZBTB7A antibody (A300-549A, Bethyl) or Rabbit IgG (B900610, Proteintech). Then the ZBTB7A binding DNA was precipitated and purified. qRT-PCR was employed to analyze the enriched DNA. Primers for E1 element F: AGGACCATGGGTGGAACAAG, R: CCTAAAGGCTGCCCTACTGTC.

### Statistical analysis

Gene expression data were analyzed using the P-values, fold changes, and ranks from the Oncomine database. Survival curves were produced by the GEPIA database. The correlation of gene expression was evaluated in the TIMER databases using Spearman's correlation analysis. Other data were evaluated using Student’s t-test. P value < 0.05 was considered statistically significant.

## Results

### The levels of ZBTB7A mRNA in UCEC and other cancers

**Fig.1:**
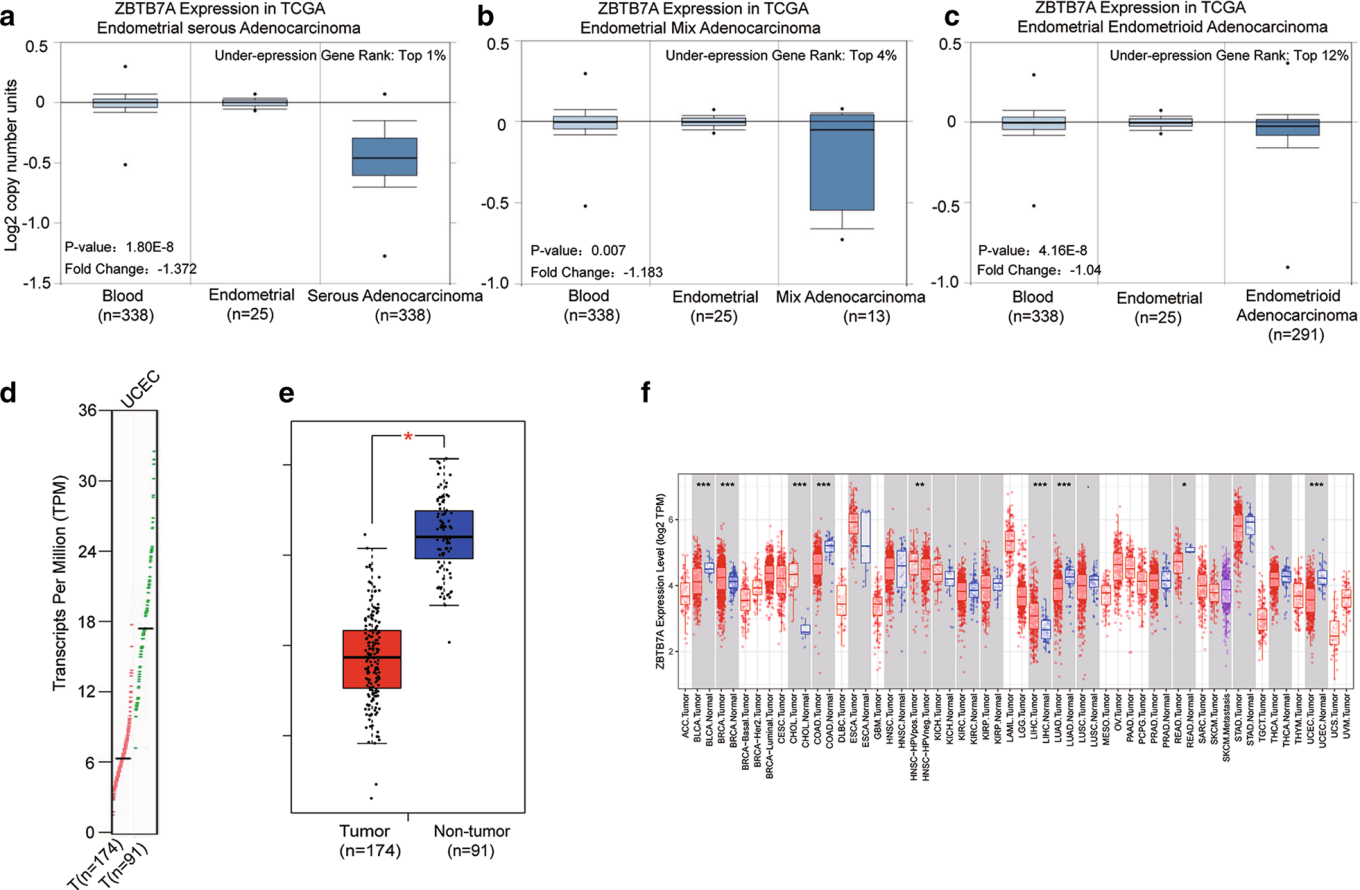
Levels of ZBTB7A mRNA are significantly lower in UCEC than in normal tissue. **a–c** ZBTB7A mRNA levels in the Endometrial Serous Adenocarcinoma, Endometrial Mixed Adenocarcinoma, and Endometrial Endometrioid Adenocarcinoma of TCGA datasets respectively. Under-expression gene rank, P values, and fold change from Oncomine 4.5 analysis. **d**, **e** ZBTB7A mRNA levels in UCEC of GEPIA datasets. **f** The ZBTB7A expression in different tumor types from the TCGA database in TIMER database. *P < 0.05, **P < 0.01, ***P < 0.001

ZBTB7A transcription levels in UCEC study were evaluated from oncomine and TIMER. The mRNA expression and DNA copy number variation (CNV) of ZBTB7A were significantly lower in UCEC tissues than in healthy tissues revealed in the Oncomine 4.5 database (P ˂ 0.01). ZBTB7A ranked within the top 20% based on mRNA expression and within the top 0.5% depended on DNA CNVs when the fold differences were within 2 (Fig. 1a–c). We compared the mRNA expression of ZBTB7A between UCEC and normal tissues of the endometrium. The results indicated that the expression level of ZBTB7A was lower in UCEC than in normal endometrium by the GEPIA dataset (https://gepia.cancer-pku.cn/) (Fig. 1d, e). Besides, The analysis of TCGA RNA-seq data using the TIMER database showed that ZBTB7A mRNA expression was also markedly downregulated in BLCA (bladder urothelial carcinoma), COAD (colon adenocarcinoma), LUAD (lung adenocarcinoma), READ (rectum adenocarcinoma) tissues in compared with the corresponding normal tissues (Fig. 1f). By contrast, ZBTB7A expression was upregulated in BRCA (breast invasive carcinoma), CHOL (cholangiocarcinoma), HNSC-HPV pos (Head and Neck squamous cell carcinoma-human papilloma virus positive), HNSC-HPV neg (Head and Neck squamous cell carcinoma-human papilloma virus negative) and LIHC (liver hepatocellular carcinoma). These data implied that downregulated ZBTB7A expression in UCEC might act as a potential diagnostic indicator.

### Sub-group analysis of multiple clinic pathological features and survival of ZBTB7A

**Fig.2:**
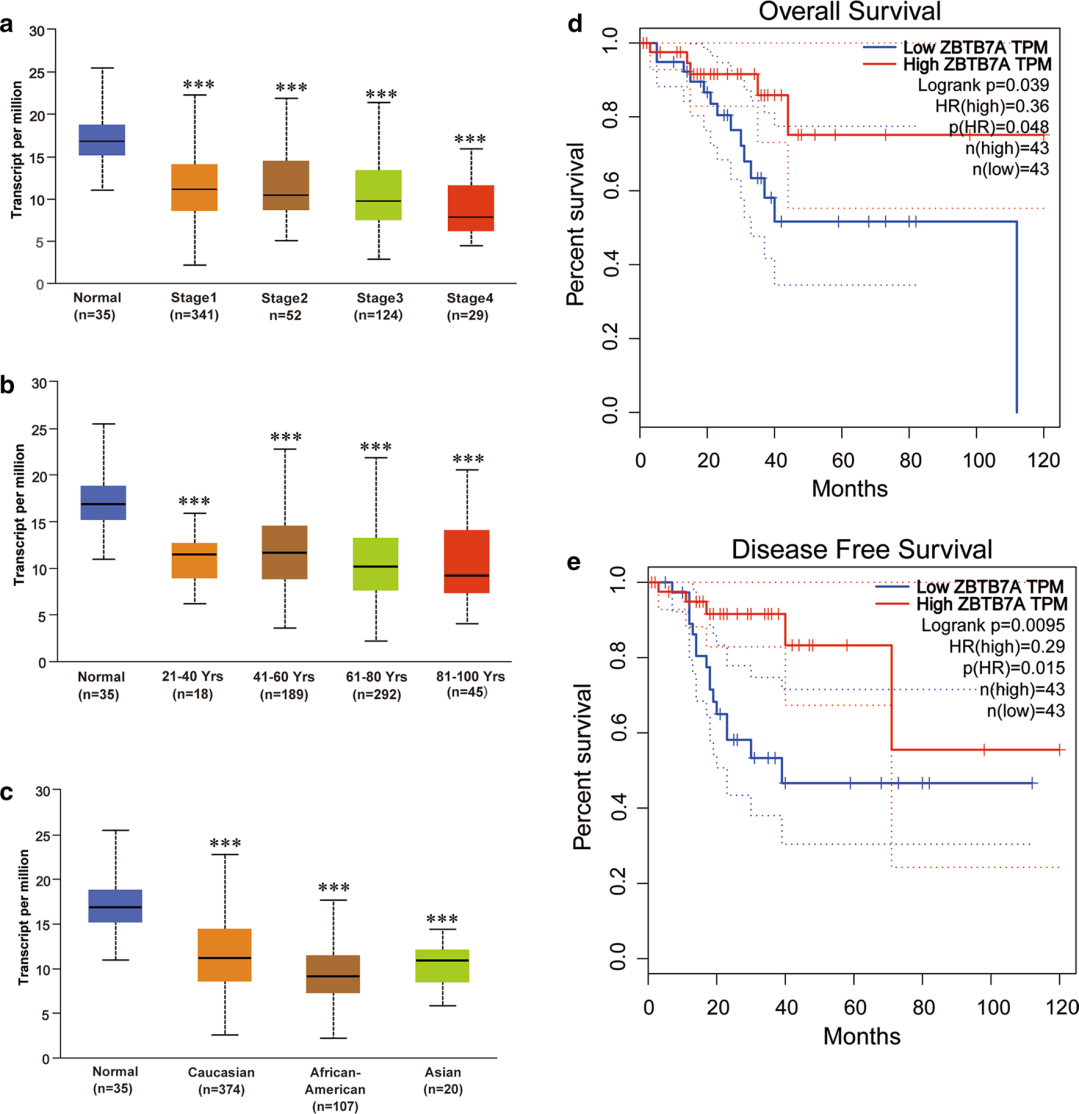
ZBTB7A level in subgroups of patients with UCEC, stratified based on disease stages, age, race (UALCAN) and survival (GEPIA). **a** Boxplots show relative expression of ZBTB7A in normal individuals or in UCEC patients in stages 1, 2, 3 or 4; (**b**) in normal individuals of any age or in UCEC patients aged 21–40, 41–60, 61–80, or 81–100 years; (**c**) in normal individuals of any ethnicity or in UCEC patients of Caucasian, African-American or Asian ethnicity. **d**–**e** Overall survival and disease free curves of ZBTB7A in UCEC in GEPIA

Further analysis of various clinic subtype features in 546 UCEC samples from the TCGA consistently established low transcription of ZBTB7A. The ZBTB7A transcription level was markedly lower in UCEC patients than in the healthy endometrium group associated with stages, age, and race (Fig. 2a–c). We further tested the clinical efficiency of ZBTB7A in UCEC. TIMER analyses uncovered that patients with downregulated ZBTB7A mRNA level harbored unfavorable overall survival (OS) ad disease free survival (DFS) (P < 0.05) (Fig. 2d, e) in UCEC. Besides, we also found that ZBTB7A mRNA expression was related to the survival in 2 cancers out of 9 cancers, including CHOL and HNSC-HPV pos (Additional file 1: Fig. S1).

### ZBTB7A genes mutation and protein interaction relationship analysis in UCEC.

**Fig. 3:**
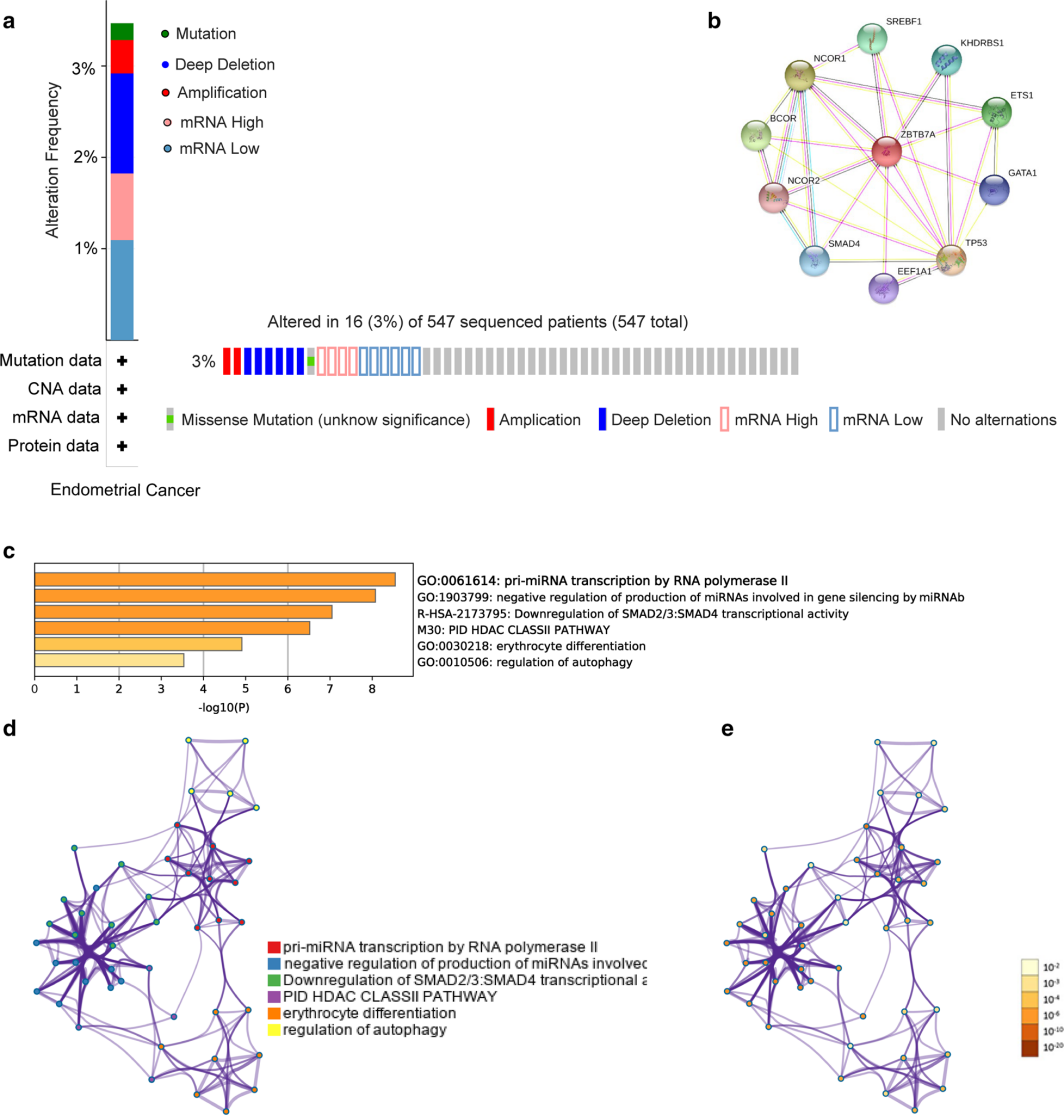
ZBTB7A gene mutation and protein interaction relationship analysis in UCEC**.** **a** ZBTB7A genes expression and mutation analysis in UCEC (cBioPortal). **b** protein–protein interactions of key genes. **c** Bar graph of enriched terms across input gene lists, colored by P-values. **d** Colored by cluster ID, where nodes that share the same cluster ID are typically close to each other. **e** Colored by P-value, where terms containing more genes tend to have a more significant P-value

In this study, the cBioPortal online tool was used for analysis of ZBTB7A alterations. ZBTB7A was altered in 36 samples out of 409 patients with UCEC (9%). Two or more aberrant changes for ZBTB7A were detected in 19 samples (3.47%) (Fig. 3a). The protein interaction relationships between key genes were analyzed using STRING, and a strong relationship between critical genes was shown in Fig. 3b.

### Enriched genes of UCEC in pathway and process enrichment analysis

For finding enriched genes in UCEC, pathway and process enrichment analysis were achieved using the following database resource: GO Biological Processes, KEGG Pathway, Canonical Pathways, Reactome Gene Sets, and CORUM. Top 6 representative enriched clusters were shown in Fig. 3c. To further catch the relationships between the terms, a subset of enriched terms had been selected and displayed as a network plot, where terms with a similarity > 0.3 were connected by edges. The network was visualized using Cytoscape, where each node represented an enriched term and colored first by its cluster ID (Fig. 3d) and then by its P-value (Fig. 3e). Specifically, the genes enriched in UCEC involved in several biological functions, such as pri-miRNA transcription by RNA polymerase II, negative regulation of production of miRNAs involved in gene silencing by miRNA, erythrocyte differentiation, regulation of autophagy, and so on.

### ZBTB7A networks of kinase, miRNA or transcription factor targets in UCEC

To further explore the targets of ZBTB7A in UCEC, we analyzed the kinase, miRNA and transcription factor target networks of positively correlated gene sets generated by GSEA (Table 1). The top 5 most significant target networks were the kinase-target networks related primarily to the kinases polo like kinase 1 (PLK1), cyclin-dependent kinase 2 (CDK2), Aurora kinase B (AURKB), cyclin-dependent kinase 1 (CDK1), and tumor-associated macrophages (ATM). In fact, PLK1, CDK1, and AURKB were significantly upregulated in tumor tissues, by contrast, ATM was markedly downregulated expressed in tumor tissues, except CDK2 (Additional file 2: Fig. S2). In addition, all these kinase genes were not significantly associated with the OS of UCEC (Additional file 2: Fig. S2). The miRNA-target network was associated with (GACTGTT) miR-212, miR-132, (AGTCTTA) miR-499, (CTGTTAC) miR-202, (TGTATGA) miR-485-3P, and (GGGATGC) miR-522. The transcription factor-target network was related mainly to the E2F transcription factor (E2F) family, including V$E2F4DP2_01, V$E2F_Q4_01, V$MAZ_Q6, GCGSCMNTTT_UNKNOWN, and KCCGNSWTTT_UNKNOWN.

**Table 1:** The Kinase, miRNA and transcription factor-target networks of ZBTB7A in UCEC (LinkedOmics)

Enriched Category	Geneset	LeadingEdgeNum	FDR
Kinase target	Kinase_PLK1	41	0
	Kinase_CDK2	112	0
	Kinase_AURKB	34	0
	Kinase_CDK1	94	0
	Kinase_ATM	63	0
miRNA target	GACTGTT, miR-212, miR-132	57	0.0091549
	AGTCTTA, miR-499	37	0.010299
	ATACCTC, miR-202	63	0.020598
	TGTATGA, miR-485-3p	57	0.020598
	ACCATTT, miR-522	62	0.025176
Transcription factor target	V$E2F4DP2_01	80	0
	V$E2F_Q4_01	69	0
	`V$MAZ_Q6	52	0
	GCGSCMNTTT_UNKNOWN	33	0.00032365
	KCCGNSWTTT_UNKNOWN	44	0.00033906

LeadingEdgeNum, the number of leading edge genes; FDR, false discovery rate from Benjamini and Hochberg from gene set enrichment analysis (GSEA); V$, the annotation found in Molecular Signatures Database (MSigDB) for transcription factors (TF)

### TPX2 and TTK in the ZBTB7A co-expression network may be targets of the E2F4 transcription factor

**Fig. 4:**
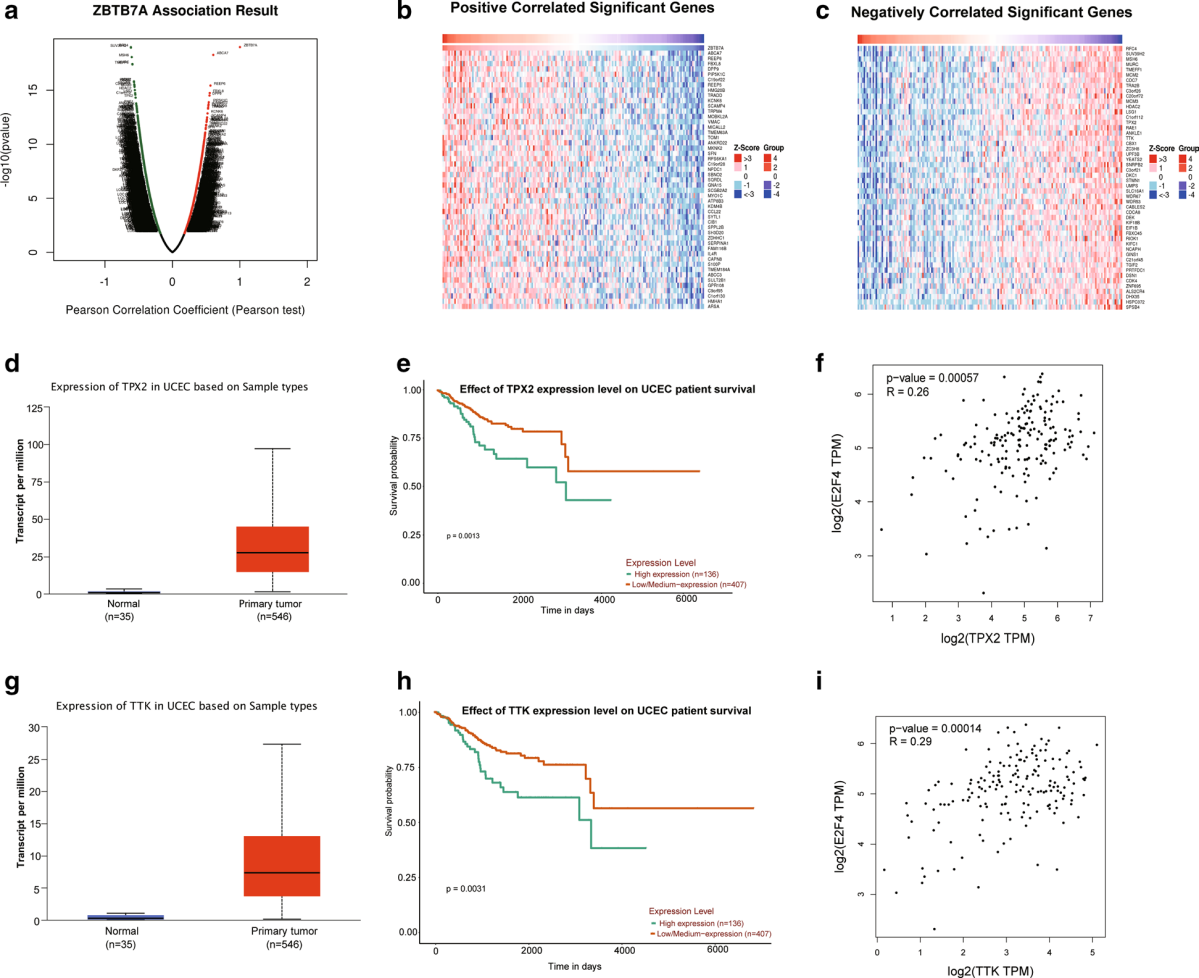
Genes differentially expressed in correlation with ZBTB7A in UCEC (LinkedOmics). **a** A Pearson test was used to analyze correlations between ZBTB7A and genes differentially expressed in UCEC. **b**–**c** Heat maps showing genes positively and negatively correlated with ZBTB7A in UCEC. Red represents positively correlated genes and green indicates negatively correlated genes. The high expression of TPX2 (**d, e**) and TTK (**g**, **h**) demonstrates unfavorable progression in UCEC, and shows positive relativity with E2F4 (**f**, **i**)

The function module of Linkedomics was used to analyze mRNA sequencing data from 176 UCEC patients in the TCGA. As shown in the volcano plot (Fig. 4a), 8768 genes (dark red dots) showed significant positive correlations with ZBTB7A, whereas 11,130 genes (dark green dots) showed significant negative correlations (false discovery rate [FDR] < 0.01). The heat map indicated that the 50 significant gene sets positively and negatively correlated with ZBTB7A (Fig. 4b, c). Among 50 negatively related genes, The expression of TPX2 (Fig. 4d, e) and TTK (Fig. 4g–h) are upregulated and related to the poor prognosis of UCEC. The level of TPX2 (Fig. 4f, R = 0.26, P-value = 0.00057) and TTK (Fig. 4i, R = 0.29, P-value = 0.00014) has direct correlation with E2F4 in UCEC. Previous studies showed that in esophageal squamous cell carcinoma, LINC00337 could recruit E2F4 to upregulate TPX2 transcription, form LINC00337/E2F4/TPX2 axis [21]. In anaplastic thyroid cancer (ATC), E2F4 overexpression inhibits the transcription of the TTK in ATC cells [22]. The above suggests that TPX2 and TTK may be the target genes of E2F4 in UCEC.

### ZBTB7A is correlated with tumor purity and immune infiltration level in UCEC

**Fig. 5:**
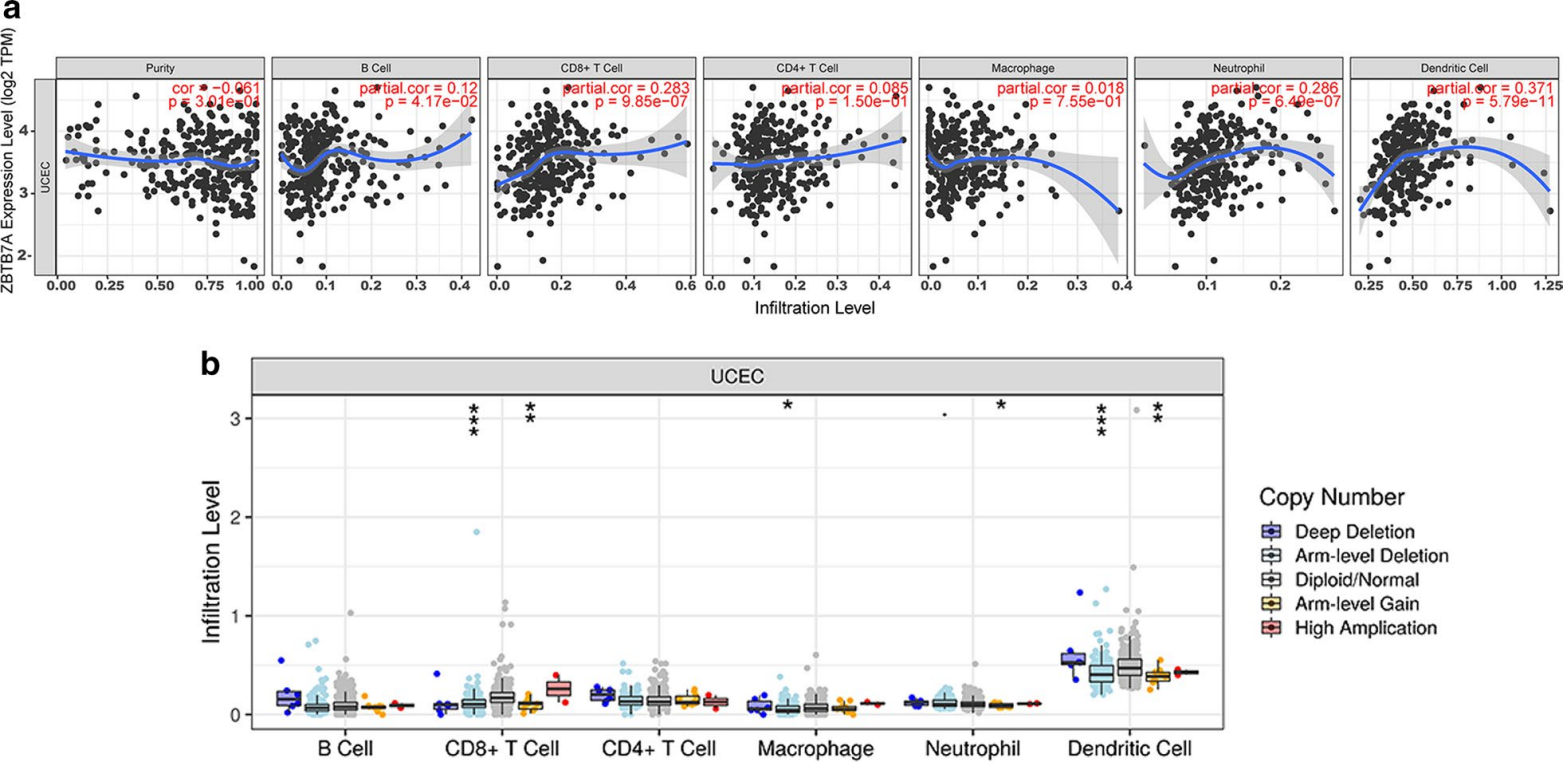
The cox proportional hazard model of ZBTB7A and six tumor-infiltrating immune cells in UCEC (TIMER). **a** ZBTB7A expression had significant positively correlations with infiltrating levels of CD8^+^ T cells, Neutrophil and Dendritic cells. **b** ZBTB7A CNV affects the infiltrating levels of CD8 + T cells, Macrophages, Neutrophils, and Dendritic cells in UCEC

We investigated whether ZBTB7A expression was correlated with immune infiltration levels in UCEC from the TIMER database. The results showed that ZBTB7A expression had no correlations with tumor purity (r = − 0.016, *P* = 2.01E−01), but powerful correlated with the immune cells infiltration levels include CD8 + T cells, neutrophils, and dendritic cells (Fig. 5a). Besides, we explored the relationship between ZBTB7A expression and the infiltrating immune cells in 9 cancer types, including BLCA, COAD, LUAD, READ, BRCA, CHOL, HNSC-HPV pos, HNSC-HPV neg, LIHC using the TIMER database. The results showed that ZBTB7A expression negatively correlated with tumor purity in 2 cancer types (LUAD, READ). Moreover, ZBTB7A expression significantly correlated with the infiltration levels of B cells in 4 cancer types (BRCA, LIHC, LUAD, and READ), CD4 + T cells in 7 cancer types (BRCA, COAD, HNSC-HPV pos, HNSC-HPV neg, LIHC, LUAD, and READ), macrophages in four cancer types (BRCA, CHOC, LIHC, and LUAD), neutrophils in six cancer types (BLCA, CHOL, COAD, HNSC-HPV pos, HNSC-HPV neg, and LUAD), dendritic cells in five cancer types (COAD, HNSC-HPV pos, HNSC-HPV neg, LIHC, and LUAD). However, ZBTB7A was not correlated with the infiltration levels of CD8 + T cells in nine cancer types (Additional file 3: Fig. S3).

Particularly, ZBTB7A CNV has significant correlations with infiltrating levels of CD8 + T cells, macrophages, neutrophils, and dendritic cells (Fig. 5b). Otherwise, ZBTB7A CNV has significant correlations with infiltrating levels of B cell in 5 cancer types (BRCA, LUAD, COAD, HNSC-HPV pos, and HNSC-HPV neg), CD8 + T cells in 4 cancer types (BRCA, COAD, HNSC-HPV pos, and HNSC-HPV neg), CD4 + T cells in 2 cancer types (BLCA and BRCA), macrophages in 4 cancer types (BLCA, BRCA, LUAD, and HNSC-HPV neg), neutrophils in 6 cancer types (BRCA, CHOL, LUAD, READ, COAD, and HNSC-HPV neg), dendritic cells in 3 cancer types (LUAD, COAD, and HNSC-HPV neg) (Additional file 4: Fig. S4).

### Correlation analysis between mRNA levels of ZBTB7A and markers of different subsets of immune cells

**Fig. 6:**
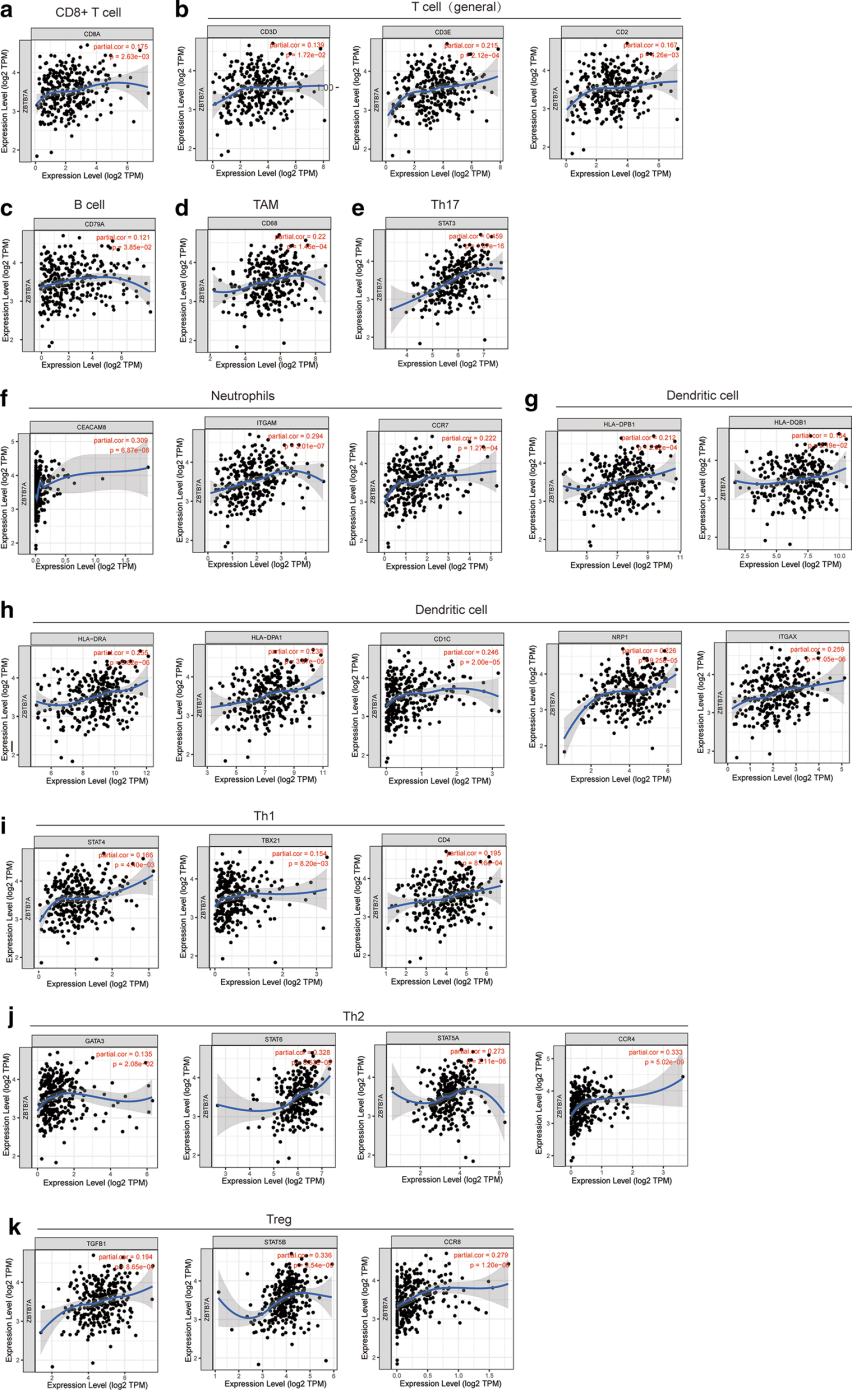
Correlation of ZBTB7A expression and the marker genes of infiltrating immune cells in UCEC (TIMER). The scatter plots showed correlation between ZBTB7A expression and the gene markers of (**a**) CD8^+^ T cell (CD8A); (**b**) T cell (general) (CD3D, CD3E, and CD2); (**c**) B cell (CD79A); (**d**) TAM (CD68); (**e**) Th17 (STAT3); (**f**) Neutrophils (CD66b, CD11b, and CCR7); (**g, h**) Dendritic cell (HLA-DPB1, HLA-DQB1, HLA-DRA, HLA-DPA1, BDCA1, BDCA4 and CD11c); (**i**) Th1 (STAT4, T-bet, and CD4); (**j**) Th 2 (GATA3, STAT6, STAT5A, and CCR4); (**k**) and Treg (TGFB1, STAT5B, and CCR8) in UCEC samples (n = 545)

Next, we investigated the relevance between ZBTB7A expression and the status of tumor-infiltrating immune cells based on the levels of immune marker gene in UCEC tissues through investigation the TIMER and GEPIA databases. The immune cells analyzed in UCEC tissues included CD8 + cell, B cells, tumor-associated macrophages (TAMs), neutrophils, and Dendritic cell. Moreover, different subsets of T cells, namely, T cell (general), T helper 1 (Th1), Th2, Th17, and regulatory T (Tregs) were also examined. Tumor purity of clinical samples influences the analysis of immune infiltration, the correlation analysis was adjusted for purity (Table. 2). Data from TIMER databases showed that ZBTB7A in UCEC tissues had a strong correlation with the marker genes from tumor-infiltrating neutrophils, dendritic cell (Fig. 6 and Table. 2). Specifically, ZBTB7A expression domonstrated noticeable interaction with the markers of specific immune cells such as neutrophils marker, CD66b (r = 0.309; P = 6.87e−08), CD11b (r = 0.294; P = 3.01e−07), and CCR7 (r = 0.222; P = 1.27e-04), Dendritic cell markers, HLA-DPB1 (r = 0.212; P = 2.63e−04), HLA-DQB1 (r = 0.134; P = 2 = e−02), HLA-DRA (r = 0.255; P = 9.82e−06), HLA-DPA1 (r = 0.238; P = 3.87e−05), BDCA1 (r = 0.246; P = 2.00e−05), BDCA4 (r = 0.226; P = 9.25e−05), and CD11c (r = 0.259; P = 7.05e−06). Besides, The expression of ZBTB7A also exhibited relevance with the marker genes of different subsets of T cells, namely, T cell (general), CD3D (r = 0.139; P = 1.72e−02), Th1 marker, STAT4 (r = 0.166; P = 4.40e−03), T-bet (r = 0.154; P = 8.20e−03), and CD4 (r = 0.195, P = 8.16e−04), Th2 marker, GATA3 (r = 0.135, P = 2.08e−02), STAT6 (r = 0.328, P = 8.53e−09), STAT5A (r = 0.273, P = 2.11e−06), CCR4 (r = 0.333, P = 5.02e−09), and Treg marker TGFβ(r = 0.194, P = 8.65e−04), STAT5B (r = 0.336, P = 3.54e−09), and CCR8 (r = 0.279, P = 1.20e−06). ZBTB7A expression did not show any significant correlation with the expression of marker genes for CD8 + T cells, B cells, TAM and Th17 cells in UCEC. These findings suggest a relation between ZBTB7A expression and immune infiltration.

**Table 2:** Correlation analysis between ZBTB7A and related markers of immune cells

Description	Gene marker	ZBTB7A (Purity)	
		Cor	P
CD8^+^ T cell	CD8A	0.175	**
	CD8B	0.105	0.074
T cell (general)	CD3D	0.139	**
	CD3E	0.215	***
	CD2	0.167	**
B cell	CD79A	0.121	*
	CD19	0.042	0.471
TAM	CD68	0.22	***
	CCL2	– 0.05	0.395
	IL10	– 0.004	0.945
Neutrophils	CD66b (CEACAM8)	0.309	***
	CD11b (ITGAM)	0.294	***
	CCR7	0.222	***
Dendritic cell	HLA-DPB1	0.212	***
	HLA-DQB1	0.134	*
	HLA-DRA	0.255	***
	HLA-DPA1	0.238	***
	BDCA1 (CD1c)	0.246	***
	BDCA4 (NRP1)	0.226	***
	CD11c (ITGAX)	0.259	***
Th1	STAT4	0.166	**
	T-bet (TBX21)	0.154	**
	CD4	0.195	***
	TNF-α (TNF)	0.041	0.484
	IFN-γ (IFNG)	0.083	0.159
Th2	GATA3	0.135	*
	STAT6	0.328	***
	STAT5A	0.273	***
	CCR4	0.333	***
	IL13	0.066	0.259
	CXCR4	– 0.039	0.508
Th17	STAT3	0.459	***
	IL17A	0.059	0.317
Treg	TGFβ (TGFB1)	0.194	***
	STAT5B	0.336	***
	CCR8	0.279	***

TAM, tumor-associated macrophage; Th: T helper cell; Treg, regulatory T cell; Cor, R value of Spearman’s correlation; Purity, correlation adjusted by purity. *P < 0.05, **P < 0.01, ***P < 0.001

### ZBTB7A represses E2F4 promoter activities in endometrial cancer cells

**Fig. 7:**
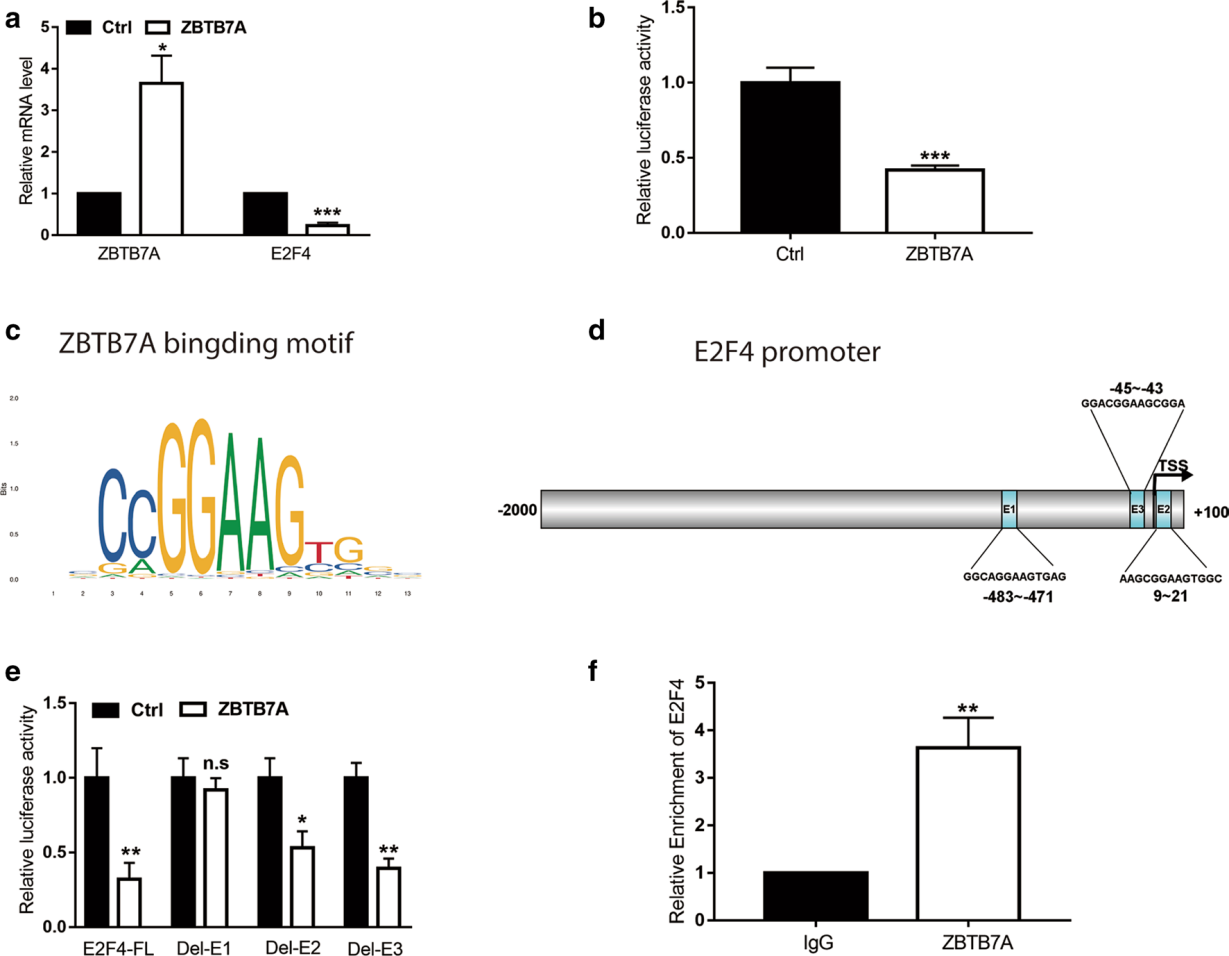
ZBTB7A can repress E2F4 transcription in endometrial cancer cells. **a** E2F4 mRNA levels with ZBTB7A overexpression in Ishikawa cells. **b** E2F4 promoter transcriptional activity was detected by Luciferase assay when cells were transfected with 300 ng ZBTB7A plasmid. **c** Schematic diagram of ZBTB7A binding motif from JASPAR Database. **d** Three prospected ZBTB7A responsive elements (E1, E2, and E3) in the E2F4 promoter part. TSS represents the transcriptional start site of E2F4. **e** Three potential ZBTB7A binding sites of the E2F4 promoter was eliminated respectively and named Del-E1, Del-E2, and Del-E3. The transcriptional activity of three depleted promoter were examined by Luciferase assay when ZBTB7A plasimid was transfected into Ishikawa cells. **f **Chromatin immunoprecipitation (ChIP) assay revealed that ZBTB7A might bind to the E1 part of E2F4 promoter in Ishikawa. The values are presented as the means ± SD from three independent experiments. *P < 0.05, **P < 0.01, ***P < 0.001

Analysis of ZBTB7A networks for transcription factor targets in UCEC, We found that ZBTB7A was associated with E2F4. Therefore, we chose Ishikawa cells to further verify the detected results from bioinformatics information. qRT-PCR demonstrated that ZBTB7A overexpression repressed E2F4 mRNA levels in Ishikawa cells (Fig. 7a). Luciferase assay indicated the E2F4 promoter (− 2000 ~ + 100) was significantly inactivated by ZBTB7A overexpression (Fig. 7b). Three potential ZBTB7A binding elements with E2F4 promoter from bioinformatics analysis (Fig. 7c–d). To examine which part was necessary for ZBTB7A mediated E2F4 expression, three predicted ZBTB7A binding sites from JASPAR database were deleted respectively. We found ZBTB7A nearly lose the ability to inhibit E2F4 transcriptional activity without the Del-E1 element (Fig. 7e), which implied the essence of E1 element for ZBTB7A binding to repress E2F4 transcription. To support our findings, chromatin immunoprecipitation (ChIP) assay was performed for E1 element. As shown in Fig. 7f, ZBTB7A could bind to E2F4 promoter and were enriched in the E1 region (− 483 ~ −471 bp).

### ZBTB7A inhibits proliferation and migration of endometrial cancer cells.

**Fig. 8:**
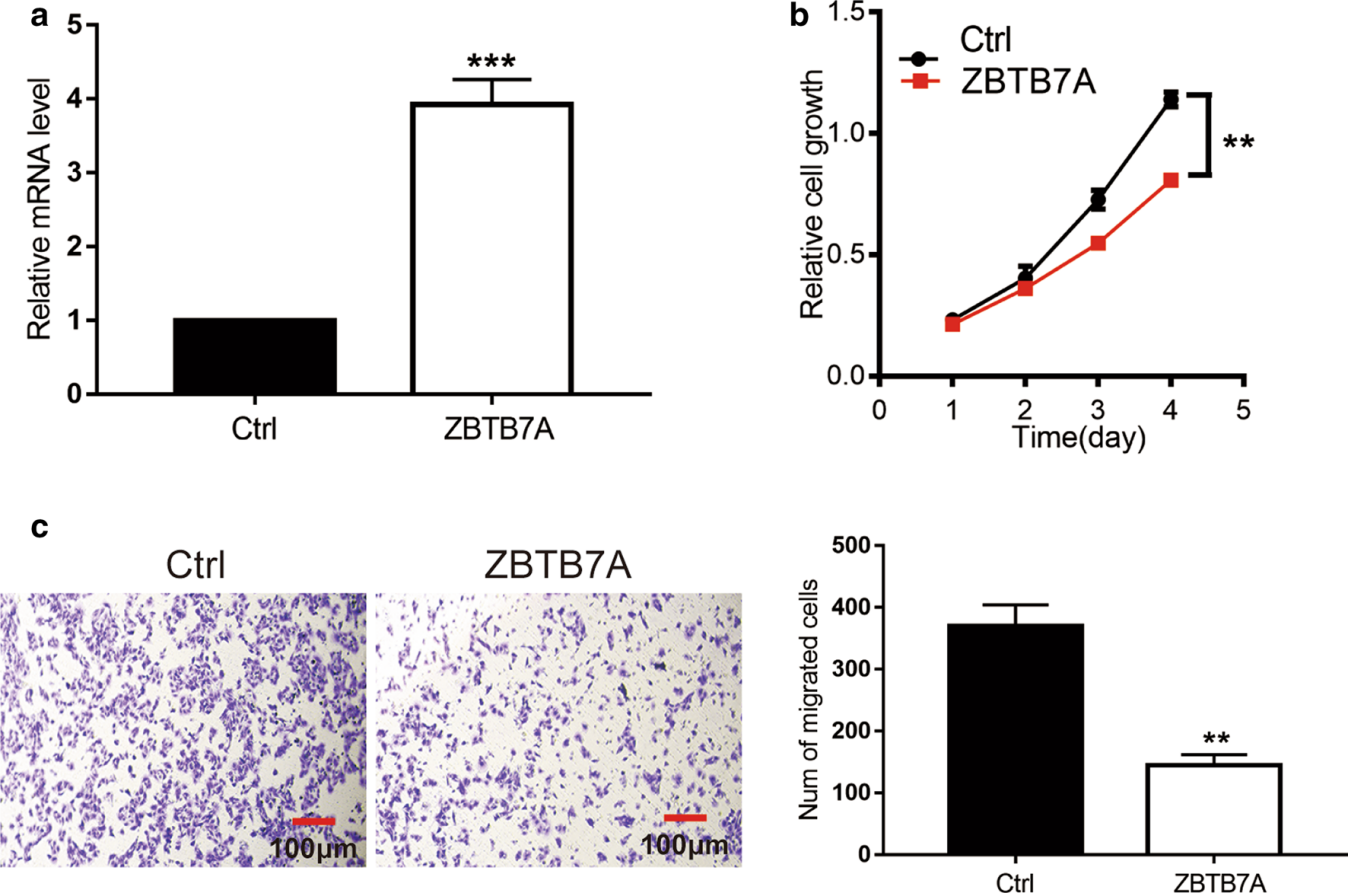
ZBTB7A inhibits proliferation and migration of Ishikawa cells. **a** The effect of plasmid pcDNA4-ZBTB7A overexpressed in Ishikawa cells. ZBTB7A inhibits growth (**b**), and migration (**c**) in Ishikawa cells

Next, we explored whether ZBTB7A affects biological function of endometrial cancer cells. ZBTB7A mRNA level was upregulated in Ishikawa cells after transfection with pcDNA4-ZBTB7A plasmid (Fig. 8a). CCK-8 assay demonstrated that upregulated ZBTB7A notablely suppressed proliferation of Ishikawa cells (Fig. 8b). Furthermore, Migration assay revealed that overexpression of ZBTB7A attenuated the ability of migration (Fig. 8c).

## Disscusion

ZBTB7A is an essential factor in regulating different aspects of human cancer. To understand more detail potential function and regulatory network of ZBTB7A in UCEC, we conducted the bioinformatics analysis of public databases to provide future research of UCEC. This study shows that ZBTB7A mRNA levels are markedly downregulated in the Endometrial Serous Adenocarcinoma, Endometrial Mixed Adenocarcinoma, and Endometrial Endometrioid Adenocarcinoma of oncomine datasets. Low ZBTB7A mRNA expression not merely correlates with unfavorable prognosis in UCEC, but also in CHOL and HNSC-HPV cancers, Furthermore, There exist a markedly relationshiop between ZBTB7A and chlinical characteristics for age, race, and tumor stage in UCEC. There was variability in the expression of ZBTB7A in different types of cancers, which may reflect differences in the data collection methods and the underlying causative mechanisms. However, the ZBTB7A expression data was consistent in UCEC tissues across various databases. These data strongly imply that ZBTB7A is a potential prognostic biomarker in UCEC. Somatic copy number variation (CNVs) often occurs in human tumors. CNVs can change the content of the genome and phenotypic differences, which may provide potential prognostic information. Our studies detect the copy number of ZBTB7A is increased and that the dominant type of ZBTB7A alteration is deep deletion, which should be responsible for the shorter survival in UCEC.

To probe the signaling events in controlling abnormal ZBTB7A expression, we tested the ZBTB7A co-expression network. Data suggest that the functional consequence of ZBTB7A mainly include pri-miRNA transcription by RNA polymerase II, negative regulation of production of miRNAs involved in gene silencing by miRNA, regulation of autophagy. These findings are consistent with the molecular pathways that result in UCEC.

We further found that ZBTB7A in UCEC is associated with a network of kinases, including CDK1, PLK1, AURKB, ATM, and CDK2. These kinases can regulate the cell cycle and mitosis, and showed differential expression, except CDK2 in UCEC. PKL1 is the primary regulator of the cell cycle [23]. In the cell cycle, PLK1 controls mitotic entry and the G2/M checkpoint [24]. AURKB plays a central role in mitosis and participates in the division of cells from G2 phase to M phase [25, 26]. The expression of the mitotic cell cycle (CDK1) was significantly suppressed by IRAK1 knockdown [27]. In UCEC, ZBTB7A may regulate cell cycle progression via interacted kinases. MicroRNAs (miRNAs) are small non-coding RNAs that often serve critical roles in human diseases. Our study identified several miRNAs that were associated with ZBTB7A. The expression of miR-212/132 may be related to the regulation of hormones and metabolism [28]. Low expression of miR-202 is associated with poor prognosis of UCEC [29]. miR-522 stimulated the progression of endometrial carcinoma by inhibiting MAOB [30]. miR-499 and miR-485-3P are associated with increased risk of cancer [31, 32]. We also found that the network of transcription factors targeted by ZBTB7A was E2F4. The role of E2F4 in the differentiation of various tissues is mainly to regulate non-cell cycle genes [33], which can directly inhibit apoptosis genes [34] and promote tumor growth by protecting cancer cells from death. We used Ishikawa cells to further verify the results that ZBTB7A inhibits the transcriptional level of E2F4 at the site of – 483 ~ -– 471 promoter and predicted that TPX2 and TTK might be the target of E2F4, which needs to be further verified in the next studies. Furthermore, increased ZBTB7A expression repress Ishikawa cells proliferation and migration obviously. Our results suggest that ZBTB7A might act through this factor to regulate cell function of UCEC.

ZBTB7A mRNA levels correlate with the different immune cell type markers in UCEC. This study demonstrates that there is a strong positive correlation between ZBTB7A expression with the infiltration of CD8 + T cells, neutrophils, and dendritic cells. This suggests that ZBTB7A plays a vital role in regulating tumor immunity, and therefore influences UCEC prognosis. We observed correlation between the levels of ZBTB7A mRNA and the expression of the T cell (general), Th1, Th2, Treg, Neutrophils, and Dendritic cell, which indicates that ZBTB7A regulates infiltration and activity of Neutrophils, and Dendritic cell. ZBTB7A expression also displays association with the markers of different subsets of T helper (Th) cells, including T cell (general) (CD2, CD3D, and CD3E), Th1 (T-bet, STAT-4, IFN-γ and TNF-α), Th2 (GATA3, STAT6, STAT5A, CCR4, IL13, and CXCR4), and Tregs (TGFβ, STAT5B and CCR8). Above all demonstrate the function for ZBTB7A in regulating tumor-infiltration of T-helper cells.

## Conclusion

Our study systematically applied public database to guide the research of ZBTB7A in endometrial cancer. Our work determine that ZBTB7A is notably downregulated in UCEC, inhibits proliferation and migration in endometrial cancer cells, serves as an indicator for favorable prognosis and immune infiltration. Besides, we verify that ZBTB7A can repress the transcription of E2F4, which may be responsible for function of ZBTB7A in endometrial cancer. In Sum, ZBTB7A could be identified as an important potential biomarker for endometrial cancer.

## Supplementary information

**Additional file1: Fig. S1**. ZBTB7A mRNA level was associated with the survival in CHOL and HNSC-HPV pos.

**Additional file 2: Fig. S2.** Expression and survival outcome of ZBTB7A-related regulators. Top 5 kinase regulators of ZBTB7A co-expressed genes. PLK1, CDK1, and AURKB were significantly higher expressed in tumor tissues, ATM was dramatically lower expressed in tumor tissues, except CDK2. In addition, all these kinase genes were not significantly associated with the overall survival of UCEC.

**Additional file 3: Fig. S3.** Correlation of ZBTB7A expression with immune infiltration levels in 9 cancers in the TIMER database.

**Additional file 4: Fig. S4**. ZBTB7A CNV affecting the distribution in various immune cells in 9 cancers in the TIMER database.

## Data Availability

The data that support the findings of this study are available from the corresponding author upon reasonable request.
