# Supplementary figures and images for: ZBTB7A, a potential biomarker for prognosis and immune infiltrates, inhibits progression of endometrial cancer based on bioinformatics analysis and experiments
Source: Cancer Cell Int. 2020 Nov 9;20:542. doi: 10.1186/s12935-020-01600-5 (PMC7654049; doi:10.1186/s12935-020-01600-5)

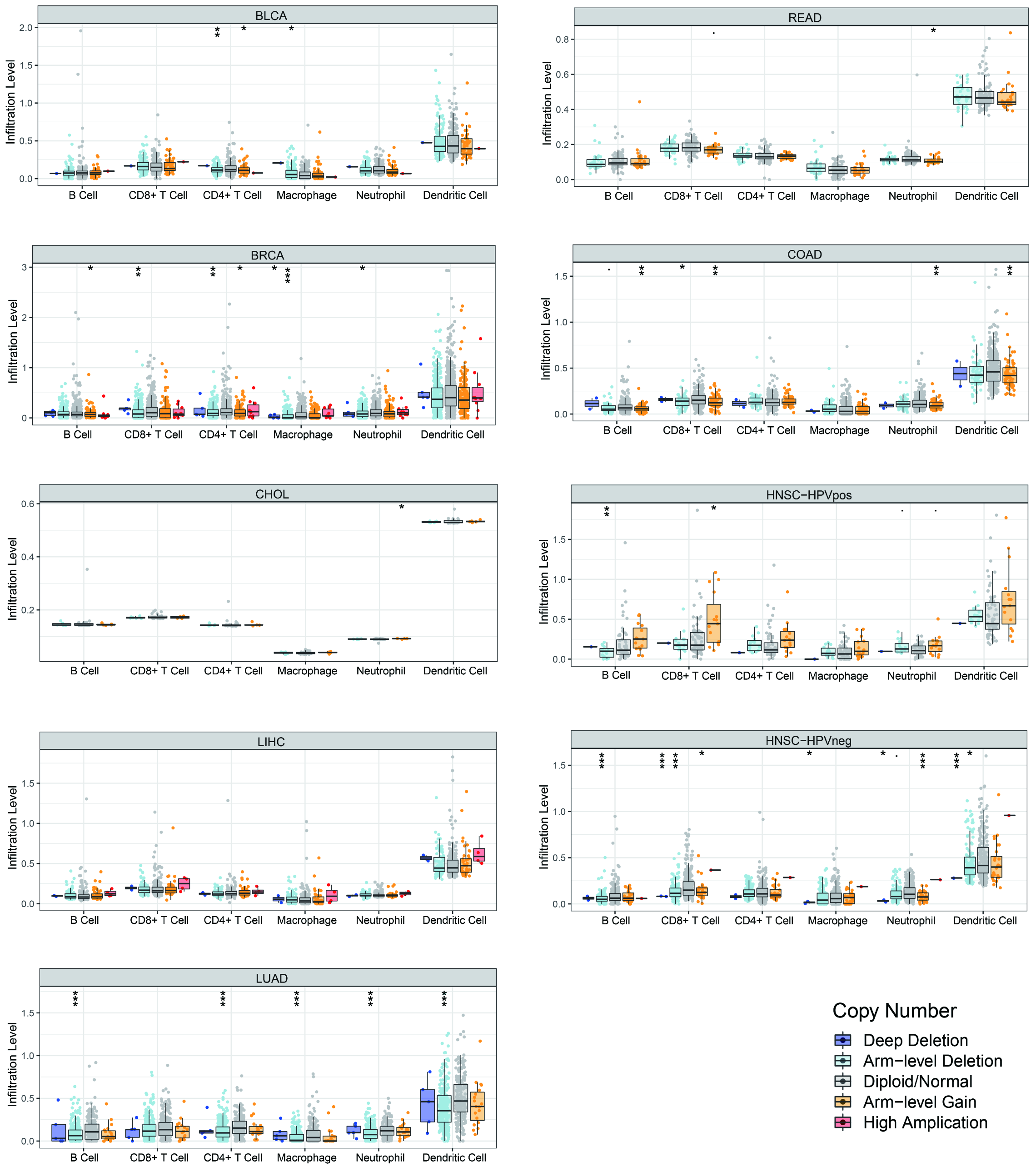

Supplement: Supplementary file 1 — Additional file1: Fig. S1. ZBTB7A mRNA level was associated with the survival in CHOL and HNSC-HPV pos. [file 12935_2020_1600_MOESM1_ESM.tif]

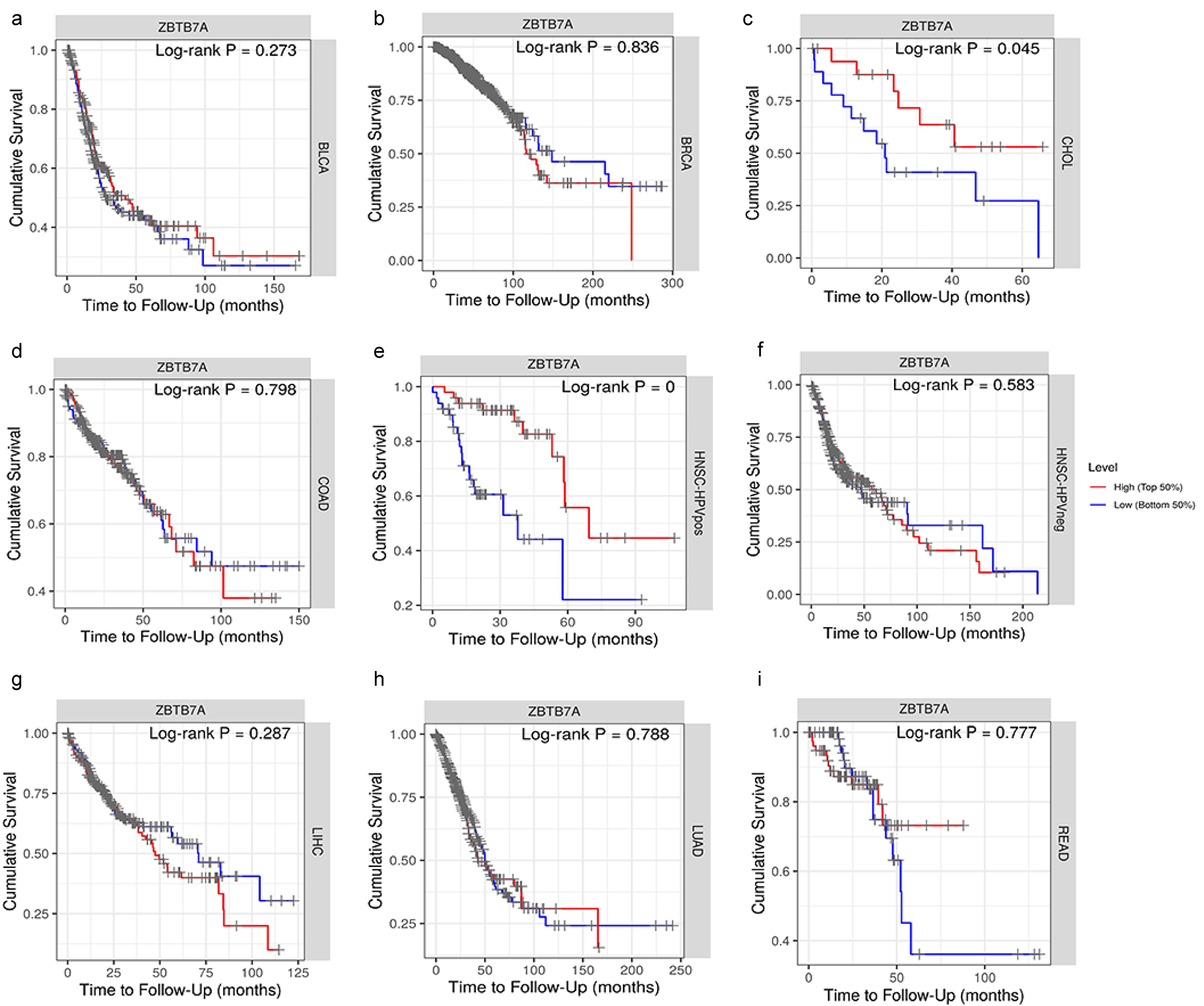

Supplement: Supplementary file 2 — Additional file 2: Fig. S2. Expression and survival outcome of ZBTB7A-related regulators. Top 5 kinase regulators of ZBTB7A co-expressed genes. PLK1, CDK1, and AURKB were significantly higher expressed in tumor tissues, ATM was dramatically lower expressed in tumor tissues, except CDK2. In addition, all these kinase genes were not significantly associated with the overall survival of UCEC. [file 12935_2020_1600_MOESM2_ESM.tif]

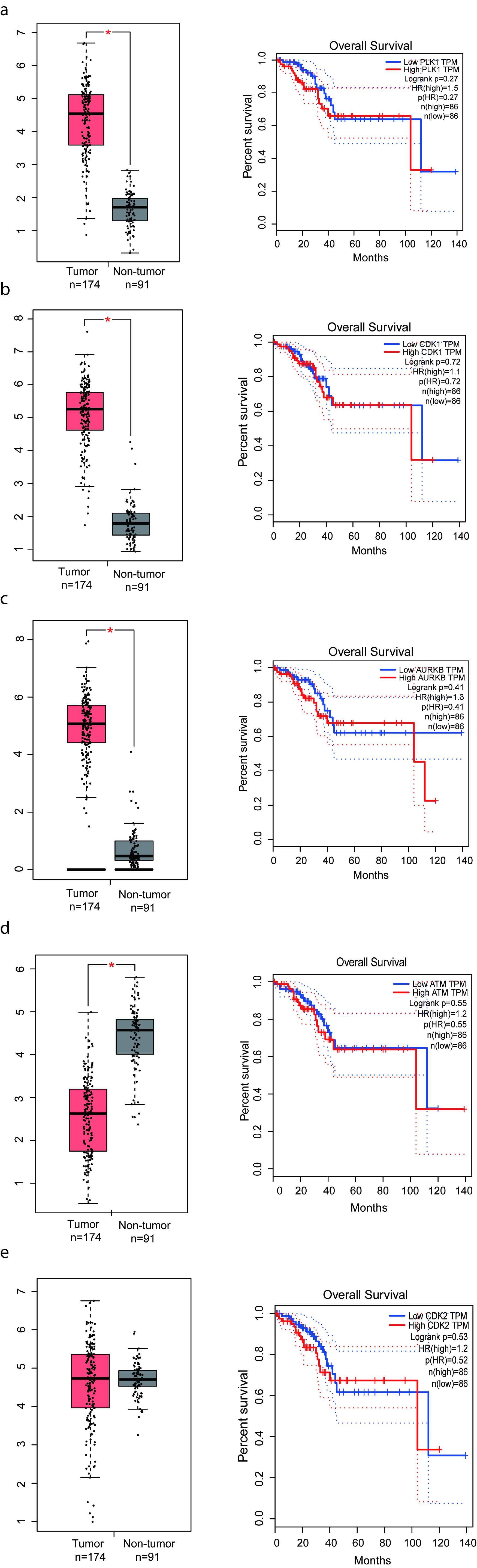

Supplement: Supplementary file 3 — Additional file 3: Fig. S3. Correlation of ZBTB7A expression with immune infiltration levels in 9 cancers in the TIMER database. [file 12935_2020_1600_MOESM3_ESM.tif]

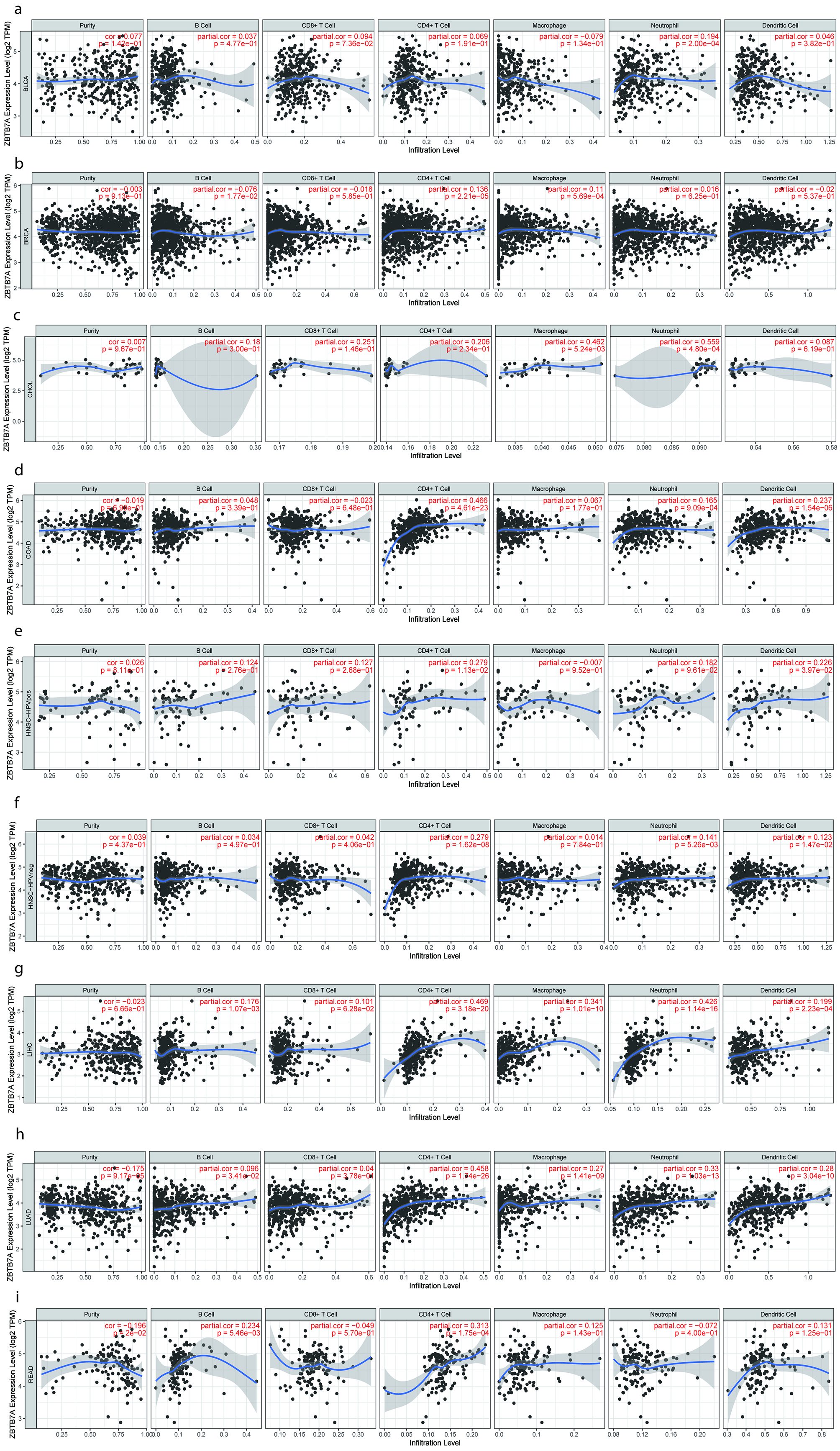

Supplement: Supplementary file 4 — Additional file 4: Fig. S4. ZBTB7A CNV affecting the distribution in various immune cells in 9 cancers in the TIMER database. [file 12935_2020_1600_MOESM4_ESM.tif]
